# Culture bag systems for clinical applications of adult human neural crest-derived stem cells

**DOI:** 10.1186/scrt422

**Published:** 2014-03-14

**Authors:** Johannes FW Greiner, Lena-Marie Grunwald, Janine Müller, Holger Sudhoff, Darius Widera, Christian Kaltschmidt, Barbara Kaltschmidt

**Affiliations:** 1 Department of Cell Biology, University of Bielefeld, Universitaetsstraße 25, Bielefeld, 33501, Germany; 2 AG Molecular Neurobiology, University of Bielefeld, Universitaetsstraße 25, Bielefeld, 33501, Germany; 3 Klinik für Hals-, Nasen- und Ohrenheilkunde, Kopf- und Halschirurgie, Staedtische Kliniken Bielefeld, Bielefeld, 33604, Germany

## Abstract

**Introduction:**

Facing the challenging treatment of neurodegenerative diseases as well as complex craniofacial injuries such as those common after cancer therapy, the field of regenerative medicine increasingly relies on stem cell transplantation strategies. Here, neural crest-derived stem cells (NCSCs) offer many promising applications, although scale up of clinical-grade processes prior to potential transplantations is currently limiting. In this study, we aimed to establish a clinical-grade, cost-reducing cultivation system for NCSCs isolated from the adult human nose using cGMP-grade Afc-FEP bags.

**Methods:**

We cultivated human neural crest-derived stem cells from inferior turbinate (ITSCs) in a cell culture bag system using Afc-FEP bags in human blood plasma-supplemented medium. Investigations of viability, proliferation and expression profile of bag-cultured ITSCs were followed by DNA-content and telomerase activity determination. Cultivated ITSCs were introduced to directed *in vitro* differentiation assays to assess their potential for mesodermal and ectodermal differentiation. Mesodermal differentiation was determined using an enzyme activity assay (alkaline phosphatase, ALP), respective stainings (Alizarin Red S, Von Kossa and Oil Red O), and RT-PCR, while immunocytochemistry and synaptic vesicle recycling were applied to assay neuroectodermal differentiation of ITSCs.

**Results:**

When cultivated within Afc-FEP bags, ITSCs grew three-dimensionally in a human blood plasma-derived matrix, thereby showing unchanged morphology, proliferation capability, viability and expression profile in comparison to three dimensionally-cultured ITSCs growing in standard cell culture plastics. Genetic stability of bag-cultured ITSCs was further accompanied by unchanged telomerase activity. Importantly, ITSCs retained their potential to differentiate into mesodermal cell types, particularly including ALP-active, Alizarin Red S-, and Von Kossa-positive osteogenic cell types, as well as adipocytes positive in Oil Red O assays. Bag culture further did not affect the potential of ITSCs to undergo differentiation into neuroectodermal cell types coexpressing β-III-tubulin and MAP2 and exhibiting the capability for synaptic vesicle recycling.

**Conclusions:**

Here, we report for the first time the successful cultivation of human NCSCs within cGMP-grade Afc-FEP bags using a human blood plasma-supplemented medium. Our findings particularly demonstrate the unchanged differentiation capability and genetic stability of the cultivated NCSCs, suggesting the great potential of this culture system for future medical applications in the field of regenerative medicine.

## Introduction

Treatment of neurodegenerative diseases as well as complex injuries, as in cancer or severe accidents, remains an important challenge in stem cell-based regenerative medicine, emphasizing the need for clinical-grade transplantation strategies. However, the relative abundances of available endogenous stem cells in their respective niches within the human body are too low to achieve significant therapeutic effects if transplanted directly into the patient without prior *in vitro* expansion [1]. Although there is a clear need for expansion steps prior to transplantation, cultivation of stem cells *in vitro* presents the inherent challenges of increasing risk of contamination, for example by transmitting infectious agents [2] or bacteria [3].

State-of-the-art stem cell culture approaches include the use of cost-intensive cleanrooms, which ensure sterility and, thus, limit the risk of contamination [4]. Culture bag systems represent a less expensive and more easily manageable alternative. In the present study, we used Afc-VueLife bags (2PF-0002, American Fluoroseal Corp., Gaithersburg, MD, USA) made of fluoroethylenepropylene (FEP), with a volume of 2 mL and two luer-ports on either side. VueLife bags are gas permeable and certified to clinical-Good Manufacturing Practice (cGMP) grade, as recommended by the Food and Drug administration (FDA) for stem cell based products [5]. Applying such Afc-FEP bags to cell expansion prior to transplantation, Lima and colleagues showed successful cultivation of cord blood cells followed by transplantation into ten patients with advanced hematological malignancies [6]. The suitability of Afc-FEP-bags to cell cultivation was further demonstrated by Krause et al. using natural killer cells [7] as well as by Hendrikx and coworkers, who applied such bags for bone marrow cells before intramyocardial transplantation [8]. Extending these promising findings, we applied Afc-FEP bags for the cultivation of neural crest-derived stem cells isolated from the adult human nose. Such inferior turbinate stem cells (ITSCs), which are suggested to possess a Schwann cell-like character endogenously [9,10], can be isolated age-independently via a minimally invasive biopsy. Cultivated *in vitro*, ITSCs show the ability to self-renew and differentiate into ectodermal and mesodermal cell types, indicating their multipotent state [10]. ITSCs may further offer the possibility to be transplanted autologously back into their donor, avoiding the need of long-term immunosuppression. Aiming for a clinical-grade expansion prior to potential future transplantations, we recently described the application of a human blood plasma (BP)-based fibrin matrix for cultivation of ITSCs, thus avoiding the use of animal sera [11]. Although the abandonment of animal sera is highly recommended by the FDA [5,12], clinically approvable production processes for ITSCs, including the use of cGMP-graded cell culture containers, is necessary for the safety of the stem cell-based products [4].

Addressing this challenge, we describe here for the first time the feasibility of a cGMP-grade Afc-FEP bag for cultivation of adult neural crest-derived human stem cells. Bag-cultured ITSCs grew three-dimensionally within the culture bags in a human BP-based fibrin matrix, demonstrating no significant changes in morphology, viability, proliferation, expression profile, genetic stability or telomerase activity in comparison to cultivation in three-dimensional cell culture flasks. Cultivation in bags did not affect the ability of ITSCs to differentiate into mesodermal or ectodermal cell types, emphasizing the potential suitability of this bag system for future clinical applications.

## Methods

### Isolation and three-dimensional-cell culture of adult human inferior turbinate stem cells

Isolation of ITSCs was performed according to [10]. Briefly, human nasal inferior turbinates were extracted via routine nasal surgery after informed consent according to local and international guidelines (Bezirksregierung Detmold/Münster). Isolation and further experimental procedures were ethically approved by the ethics commission of the Ärztekammer Westfalen-Lippe and the medical faculty of the Westfälische Wilhems-Universität (Münster, Germany) (approval reference number 2012-15-fS). Enzymatic dissociation using Dispase II (Sigma-Aldrich, Taufkirchen, Germany; 0.55 U/mg) for 12 hours at 4°C was subsequently followed by mechanical disintegration and further enzymatic digestion with collagenase NB4 (1.17 U/mL; SERVA Electrophoresis, Heidelberg, Germany) for two hours at 37°C.

ITSCs were precultivated in (Dulbecco’s) modified Eagle’s medium/Ham’s F-12 (1:1) (DMEM/F-12; Biochrom, Berlin, Germany) with penicillin and streptomycin (P/S) (0.1 mg/ml penicillin, 100 U/ml streptomycin; PAA, Pasching, Austria), amphotericin B (AB) (0.25 mg/ml; PAA), L-glutamine (L-Glu) (200 mM; Sigma-Aldrich), epidermal growth factor (EGF; 20 ng/mL; R&D Systems, Wiesbaden, Germany), basic fibroblast growth factor (bFGF-2; 40 ng/mL; lab-made) and B27 supplement [13], hereinafter referred to as ‘standard medium,’ as well as additional heparin (0.5 U/ml, Sigma-Aldrich) at 37°C, 5% CO_2_ and 5% O_2_ using a humidified incubator (Binder, Tuttlingen, Germany) and low adhesion T25 tissue culture flasks (Greiner Bio-One, Frickenhausen, Germany). After observing primary neurosphere formation, ITSCs were harvested by centrifugation at 300 × g and dissociated with collagenase NB4 (SERVA Electrophoresis) for two hours at 37°C.

For three-dimensional-cell culture, harvested ITSCs were cultivated in standard medium supplemented with clinically accredited therapeutic human blood plasma (BP) (Institut für Laboratoriums- und Transfusionsmedizin, Bad Oeynhausen, Germany) according to Greiner *et al*. [11] using a 25 cm^2^-Tissue Culture Flask or 22.1 cm^2^-Tissue Culture Dish (TPP, TechnoPlasticProcducts, Trasadingen, Switzerland). Passaging was performed as described above.

### Cultivation of ITSCs in Afc-FEP bags

For bag culture, ITSCs isolated as described above were harvested using collagenase NB4 (SERVA Electrophoresis) for two hours at 37°C followed by centrifugation at 300 × g. A defined amount of ITSCs was placed in 2 ml standard medium comprising 10% clinically accredited therapeutic human BP (Institut für Laboratoriums- und Transfusionsmedizin). Stem cell-containing medium was subsequently filled into 2 ml Afc-FEP bags 2PF-0002 (in the following referred to as ‘cell culture bag’) (Afc, American Fluoroseal Corp.) using syringes (B. Braun Melsungen AG, Melsungen, Germany) through a Luer port at the side of the bag. Cultivation was performed at 37°C, 5% CO_2_ and 5% O_2_ in a humidified incubator (Binder).

Bag-cultured ITSCs were fed at least every two days by removal of liquid medium supernatant comprising the digested BP-matrix via a syringe (B. Braun Melsungen AG) through the Luer ports. Alternatively, whole culture bags were prepared for centrifugation by folding at one end followed by placing them into 50 ml Falcon tubes (fold down to the bottom). Tubes were centrifuged at 300 × g for 10 minutes, leading to the formation of cell pellets at the respective end of the bag, which allowed removal of medium supernatant by a syringe (B. Braun Melsungen AG) through the opposite Luer ports. Removal of medium was subsequently followed by injection of standard medium comprising 10% BP (Institut für Laboratoriums- und Transfusionsmedizin) up to a total volume of 2 ml.

For passaging, ITSCs were harvested by centrifugation as described above and digested within the culture bags by adding 2 ml collagenase NB4 (SERVA Electrophoresis) for one hour at 37°C followed by a second centrifugation step. Alternatively, liquid medium supernatant was removed as described above followed by the addition of collagenase NB4 for one hour at 37°C and centrifugation. Harvested ITSCs were resuspended in 500 μl standard medium and injected in a new culture bag via syringe followed by addition of standard medium and 10% BP up to a total volume of 2 ml. Cultivation was performed at 37°C, 5% CO_2_ and 5% O_2_ in a humidified incubator (Binder).

### Transfection of cultivated ITSCs

ITSCs were cultivated under standard conditions, digested with collagenase NB4 (SERVA Electrophoresis) and harvested via centrifugation. Afterwards, ITSCs were transfected with 1 μg pmax GFP (Amaxa Biosystems, Lonza Group AG, Basel, Switzerland) using Amaxa rat NSC-Nucleofector Kit (Amaxa Biosystems) and Nucleofector II device (Amaxa Biosystems) according to the manufacturer’s guidelines.

Immediate addition of 5 ml DMEM/F-12 (Sigma-Aldrich) was followed by centrifugation for 10 minutes at 300 × g. Transfected ITSCs were suspended in 2 ml standard medium supplemented with 10% BP and injected into Afc-FEP bags 2PF-0002 (Afc) using a syringe (B. Braun Melsungen AG). Cultivation was performed at 37°C, 5% CO_2_ and 5% O_2_ for 48 hours in a humidified incubator (Binder). Live imaging of GFP-ITSCs was done via optical sectioning (Z-Stack) using confocal laser scanning microscopy (excitation wavelength: 488 nm, LSM 510, Carl Zeiss, Jena, Germany), while ZEN software (Carl Zeiss) was subsequently applied for three-dimensional reconstruction.

### Proliferation assay and vitality measurement of ITSCs

A defined number of ITSCs was cultured in Afc-FEP bags 2PF-0002 (Afc) or 9.2 cm^2^ Tissue Culture Dishes (TPP) in standard medium supplemented with 10% BP for five days as described above. For determination of total cell numbers, counting was performed after digestion with collagenase NB4 (SERVA Electrophoresis) and harvesting at 300 × g, in a Cellometer Auto T4 device (Nexcelom Bioscience, Lawrence, MA, USA). The vitality of the cultured cells was assessed using trypan blue staining (Sigma-Aldrich) according to the manufacturer’s guidelines followed by measurement with a Cellometer Auto T4 device (Nexcelom Bioscience). Doubling time was calculated according to the formulas (doubling time = ln(2)/growth rate) and [(ell amount = cell concentration (t = 0)*e^growth rate*time^). Statistical analysis of cell proliferation and vitality was performed with Graph Pad Prism (GraphPad Software, San Diego, CA, USA).

### Reverse transcription PCR

Total RNA was isolated using TRI Reagent (Sigma-Aldrich) or innuPREP RNA mini Kit (Biometra GmbH, Analytik Jena AG, Jena, Germany) according to the manufacturers’ guidelines followed by assessment of RNA-concentration and quality via Nanodrop UV spectrophotometry. For subsequent cDNA synthesis, the First Strand cDNA Synthesis Kit (Fermentas, St. Leon-Rot, Germany) or qScript cDNA synthesis Kit (Quanta Biosciences Inc., Gaithersburg, MD, USA) were applied according to the manufacturers’ guidelines. PCR was performed using GoTaq DNA polymerase (Promega, Madison, WI, USA) as indicated by the manufacturer, including application of 10 mM dNTPs (New England Biolabs, Frankfurt am Main, Germany) and 0.5 μM primers (see Table 1) (Metabion, Martinsried, Germany). Water was used instead of cDNA for negative controls.

**Table 1:** Primer sequences

**Target**	**Forward primer**	**Reverse primer**
cMyc	aggagacatggtgaaccagagt	agcctgcctcttttccacagaaac
Sox2	aaccccaagatgcacaactc	gcttagcctcgtcgatgaac
p75	tgagtgctgcaaagcctgcaa	tctcatcctggtagtagccgt
Nestin	cagcgttggaacagaggttg	gctggcacaggtgtctcaag
GAPDH	ctgcaccaccaactgcttag	gtcttctgggtggcagtgat
Osteocalcin	ctcacactcctcgccctatt	cgcctgggtctcttcactac
Osteopontin	actgattttc ccacggacct	cattcaactcctcgctttcc

### Flow cytometric measurement of DNA content

For cell cycle analysis, the DNA content of ITSCs was measured with CyStain PI absolute T-Kit (PARTEC, Münster, Germany) according to the manufacturer’s guidelines after at least three weeks of bag- or three-dimensional-culture. PI-staining was analyzed using a CyFlow space flow cytometer (PARTEC), while data analysis was assessed using FlowJo Software (TreeStar, Olten, Switzerland). Doublet discrimination was done by gating FL3-A- versus FL3-W signals [14].

### Telomerase activity measurement

Telomerase activity of bag cultured ITSCs was analyzed using the real-time Q-TRAP assay described in [15]. Briefly, ITSCs cultivated in bags were harvested via collagenase treatment as described above followed by centrifugation (3,000 × g, five minutes) and lysis using NP-40 lysis buffer [15] for 30 minutes. After centrifugation at 16,000 × g for 20 minutes at 4°C, protein concentrations were assessed via Nanodrop UV spectrophotometry. For real-time Q-TRAP, the reaction mixture was prepared by adding 2 μl protein lysate to 12 μl PERFECTa SYBR Green Super Mix (Quanta Biosciences Inc.), 9 μl H_2_O, 1 μl ACX primer (5′-GCGCGGCTTACCCTTACCCTTACCCTAACC-3′, 100 ng/μl, Sigma-Aldrich) and TS primer (5′-AATCCGTCGAGCAGAGTT-3′, 100 ng/μl, Sigma-Aldrich). Prior to qPCR, reaction mixtures were incubated for 30 min at 25°C to allow substrate extension by telomerase. Subsequent qPCR reactions were performed according to guidelines of PERFECTa SYBR Green Super Mix Kit (Quanta Biosciences Inc.) and assayed with Rotor Gene 6000 (QIAGEN, Hilden, Germany). All reactions were performed in triplicate; U251 cells cultivated as described in [16] served as a positive control. Respective Ct-values^−1^ normalized to applied protein concentrations served for calculating relative telomerase activity of ITSCs as percentage telomerase activity to positive control (positive control equal to 100% telomerase activity). Statistical analysis was performed with Graph Pad Prism (GraphPad Software).

### Directed osteogenic differentiation assay

For directed osteogenic differentiation, bag- and three-dimensional-cultured ITSCs were harvested as described above and 3 × 10^3^ cells/cm^2^ were plated on 12-well TPP tissue culture plates (TPP) in DMEM (Sigma-Aldrich) containing 10% FCS (Sigma-Aldrich, lot: 126 K3398). ITSCs were cultivated at 37°C and 5% CO_2_ for 48 hours. Afterwards, medium was replaced by osteogenic differentiation medium including DMEM (Sigma-Aldrich) supplemented with 10% FCS, 100 nM dexamethasone (Sigma-Aldrich), 10 mM β-glycerophosphate (Sigma-Aldrich) and 0.55 mM L-ascorbic acid-2-phosphate (Sigma-Aldrich). Feeding was performed every three to four days with fresh osteogenic differentiation medium. ALP activity was assessed after 9 days of differentiation, while RT-PCR-analyses as well as Alizarin Red S and Von Kossa staining were applied after differentiation for 21 days.

### Detection of alkaline phosphatase-activity

The ALP activity of ITSCs precultivated as bag- and three dimensional-culture ITSCs was measured after nine days of osteogenic differentiation. Differentiated ITSCs were fixed with 4% PFA (Paraformaldehyde) followed by staining using a freshly prepared staining solution comprising two parts Fast Red Violet (Sigma-Aldrich), one part Naphtol AS-MX phosphate alkaline solution (Sigma-Aldrich) and two parts ddH_2_O. The staining solution was incubated for 15 minutes under exclusion of light followed by determination of ALP activity via light microscopy using an AMG EVOS xl microscope (PeqLab Biotechnology).

### Alizarin Red S and Von Kossa staining for assessment of osteogenic differentiation

Alizarin Red S and Von Kossa stainings were applied after 21 days of osteogenic differentiation of bag- and three-dimensional-cultivated ITSCs. For Alizarin Red S staining, fixation with 4% PFA for 15 minutes was followed by washing with PBS (1x) and subsequent staining using 1% Alizarin Red Solution (Sigma-Aldrich) for 5 minutes. Von Kossa staining was performed after fixing differentiated ITSCs with 4% PFA for 15 minutes followed by washing with ddH_2_O. After application of 5% silver nitrate in ddH_2_O (Fluka Chemie, Buchs, Switzerland) for 60 minutes under UV-light and subsequent washing with ddH_2_O, 5% sodium thiosulfate (Fluka Chemie) was applied for three minutes at room temperature. The third washing step was followed by staining with 0.1% nuclear fast solution comprising 5% aluminum sulfate and 0.1% nuclear fast red (Merck KGaA, Darmstadt, Germany) dissolved in ddH_2_O for five minutes. The final washing was performed using 100% ethanol (Sigma-Aldrich) and two times using 96% ethanol. Imaging of Alizarin Red S and Von Kossa stained ITSCs was done via light microscopy using AMG EVOS xl microscope (PeqLab Biotechnology).

### Adipogenic differentiation assay

ITSCs pre-cultured in cell culture bags (Afc) or three-dimensionally as described above were plated at a density of 4 × 10^3^ cells per well in 12-well TPP tissue culture plates (TPP) and cultivated for 48 hours in DMEM (Sigma-Aldrich) containing 10% FCS (Sigma-Aldrich, lot: 126 K3398). Afterwards, the medium was replaced by DMEM comprising 10% FCS (Sigma-Aldrich), 1 μM dexamethasone (Sigma-Aldrich), 2 μM insulin (Sigma-Aldrich), 500 μM 3-isobutyl-1-methylxanthine (Sigma-Aldrich) and 200 μM indomethacin (Sigma-Aldrich). ITSCs were cultivated up to 28 days under differentiation conditions, while medium was changed every three days. To assess adipogenic differentiation, ITSCs were fixed with 10% PFA for 60 minutes followed by staining with freshly prepared Oil Red O solution (Sigma-Aldrich) (0.5% Oil Red O in propanol) for two hours.

### Directed neuronal differentiation

For neuronal differentiation, three-dimensional- or bag-cultured ITSCs were seeded in 12-well TPP tissue culture plates (TPP) at a density of 1×10^5^ cells/well and cultivated in DMEM (Sigma-Aldrich) containing 10% FCS (Sigma-Aldrich, lot: 126 K3398). After 48 hours, 1 μM dexamethasone (Sigma-Aldrich), 2 μM insulin (Sigma-Aldrich), 500 μM 3-isobutyl-1-methylxanthine (Sigma-Aldrich), 200 μM indomethacin (Sigma-Aldrich) and 200 mM ethanol were added to the medium to induce neuronal differentiation. Medium was additionally supplemented with 0.5 μM retinoic acid and N2 supplement after seven days of differentiation, while medium change was performed twice a week by replacing half of the medium volume. After 28 days, immunocytochemical stainings and vesicle recycling assay were performed.

### Immunocytochemistry of differentiated ITSCs

For immunocytochemistry, ITSCs were cultivated under neuronal differentiation conditions for 28 days. After fixation using 4% phosphate buffered 4% PFA, ITSCs were permeabilized with 0.02% Triton X-100 (Sigma-Aldrich) in PBS for 30 minutes at room temperature. Blocking was performed using 5% of the appropriate normal serum followed by addition of the primary antibodies anti-β-III-Tubulin (1:300, Promega) and anti-microtubule-associated protein 2 (MAP2, 1:100, Merck Millipore, Billerica, MA, USA) for two hours. Secondary fluorochrome-conjugated antibodies Alexa Fluor® 488 donkey anti-rabbit IgG (1:300, Invitrogen) and anti-mouse (1:300, Invitrogen) were applied for one hour at room temperature under exclusion of light. Nuclear counterstaining was performed using 4',6-diamidino-2-phenylindole (DAPI, 0.5 μg/ml; Sigma-Aldrich) for 15 minutes. Fluorescence imaging was performed using confocal laser scanning microscopy (LSM 510 or LSM 780, Carl Zeiss).

### Vesicle recycling

To assess the capabilitiy of ITSCs differentiated for 28 days in neuronal induction medium for synaptic vesicle recycling, FM1-43FX Lipophilic Styryl Dye (Life Technologies, Molecular Probes, Paisley, UK) was applied according to the manufacturer’s guidelines. Subsequent washing of the cells using Hank’s balanced salt solution (HBSS) (Sigma-Aldrich) was followed by staining for 90 seconds with FM1-43FX dye containing 75 mM KCl. Imaging was performed using fluorescence microscopy (AxioVert, Carl Zeiss).

## Results

### Afc-FEP cell culture bags are suitable for three-dimensional-cultivation of human ITSCs

**Figure 1:**
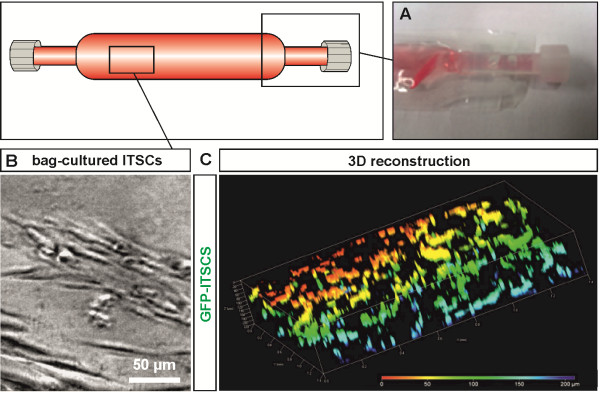
**ITSCs grow three-dimensionally in clinical grade culture bags. (A)** Afc-FEP bag containing ITSCs in medium supplemented with human blood plasma. **(B)** Phase contrast microscopy image of bag-cultured ITSCs revealing characteristic morphology comprising long-shaped cell bodies. **(C)** Confocal laser scanning microscopy analyses (Z-sectioning) followed by three-dimensional-reconstruction showed three-dimensional-growth of bag-cultured ITSCs previously transfected with GFP. FEP, fluorinated ethylene propylene; ITSCs, inferior turbinate stem cells.

In order to investigate the potential suitability for ITSC-expansion, Afc-FEP bags were filled with stem cell-containing medium supplemented with human BP (Figure 1A). Subsequent formation of a BP-based fibrin matrix as well as successful growth of ITSCs was observable within the bags, wherein ITSCs revealed characteristically long-shaped cell bodies (Figure 1B) [10,11]. To further elucidate the distribution of ITSCs growing within the cell culture bags, ITSCs were transiently transfected with GFP. GFP-signals were determined by confocal laser scanning microscopy using optical sectioning (Z-stack) followed by subsequent three-dimensional-reconstruction. ITSCs were found to be localized within all three-dimensions of the fibrin matrix, suggesting homogenous, three-dimensional-growth within the culture bags (Figure 1C).

### Bag culture does not impair viability and proliferative capability of ITSCs

**Figure 2:**
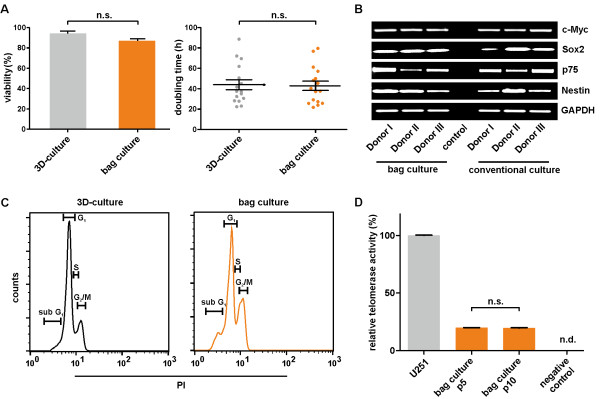
**Bag-culture does not affect vitality, expression profile, DNA content and telomerase activity of ITSCs. (A)** The amount of trypan blue-negative ITSCs (viability) and total cell numbers (doubling time) during bag- and three-dimensional-culture reveals no significant changes in vitality and proliferation (biological triplicates, *P* <0.05 is considered statistically significant; unpaired t-test, two-tailed, confidence interval: 95%; dots represent doubling times of ITSCs derived from separate donors). **(B)** Reverse transcription PCR analysis revealing unchanged expression of ITSC-specific neural crest and stemness transcripts in bag-cultivated ITSCs. **(C)** Representative flow cytometric analyses of PI-stained ITSCs after three weeks of bag- or three-dimensional-culture. Bag-cultured ITSCs exhibited a characteristic DNA content for diploid cells without any signs of polyploidy, although showing increased signals for apoptotic (sub G1) and mitotic cells (G2/M). **(D)** Real time Q-TRAP analysis depicting telomerase activity of ITSCs cultivated in bags over five passages (p5) and no significant changes during subcultivation up to passage 10 (p10) (technical triplicates, *P* <0.05 is considered statistically significant; unpaired t-test, two-tailed, confidence interval: 95%). U251 human astroglioma tumor cells served as positive control, negative control lacked template, n.d.: not detectable. ITSCs, inferior turbinate stem cells; PI, propidium iodide.

As it is crucial to ITSC-cultivation that fast expansion occurs, we investigated their viability and ability to proliferate during bag-culture. Here, trypan blue staining revealed no significant changes in viability between bag- (94.2% ± 3.56) and three-dimensional-cultured (86.98% ± 3.39) ITSCs (Figure 2A, left diagram). Proliferation of ITSCs during bag culture was determined by assessing total cell numbers of ITSCs from three donors over a defined period of time. Bag-cultured ITSCs demonstrated no significant change in doubling time (35.76 hours ± 2.47) in comparison to three-dimensional-cultures (38.98 hours ± 10.04) (Figure 2A, right diagram).

### ITSCs maintain their expression profile and genetic stability during bag-culture

Determining potential influences of bag cultivation on the expression profile of ITSCs, the messages of respective stemness and neural crest markers were assessed using RT-PCR. In comparison to three-dimensional culture, ITSC-populations derived from three donors revealed unchanged expression of the stemness markers c-Myc and Sox2 after bag cultivation. We further observed the messages of neural crest markers p75 and Nestin in three-dimensional- and bag-cultivated ITSCs, evidencing a maintained ITSC-characteristic expression profile during bag cultivation (Figure 2B).

As *in vitro* cultivation steps may be further associated with potential changes in ploidy [17], the genetic stability of ITSCs was determined after three weeks of bag cultivation. Propidium iodide (PI) staining followed by subsequent flow cytometric analysis demonstrated typical DNA content for diploid cells in bag-cultivated cells. We observed no signs of polyploidy, as indicated by the lack of detectable PI signal above the G2/M peak. Notably, ITSCs cultivated in bags showed elevated signals for apoptotic (sub G1) as well as mitotic cells (G_2_/M) in comparison to three-dimensional culture (Figure 2C).

### Subculture of ITSCs in bags does not affect their telomerase activity

Since expansion of adult human stem cells *in vitro* is broadly associated with telomere length-dependent aging [18], we investigated telomerase activity in ITSCs during bag subculture using real time Q-TRAP analysis [15]. As depicted in Figure 2D, ITSCs cultivated in bags over ten passages showed no significant changes in relative telomerase activity (19.60 ± 0.68% of control U251 human astroglioma tumor cells) compared to early passage bag cultures (19.84 ± 0.36%).

### ITSCs cultivated in bags show efficient osteogenic differentiation capability in a directed differentiation assay

**Figure 3:**
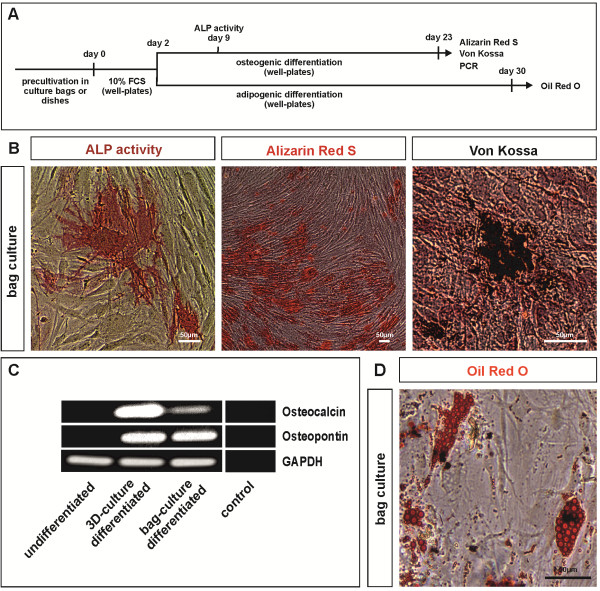
**Bag-cultured ITSCs keep their potential to undergo mesodermal differentiation. (A)** Schematic overview of directed osteogenic and adipogenic differentiation assays. **(B)** Representative phase contrast microscopy images (biological triplicate) revealing ALP-activity (left panel) as well as Alizarin Red S-(middle panel) and Von Kossa-positive (right panel) Ca^2+^-deposition of ITSCs cultivated under osteogenic differentiation conditions after bag culture. **(C)** RT-PCR analyses show expression of characteristic osteogenic markers osteocalcin and osteopontin in bag-cultured ITSCs after directed osteogenic differentiation. GAPDH served as loading control. **(D)** Representative phase contrast microscopy images (biological triplicate) exhibiting the presence of Oil Red O-positive lipid droplets within adipogenically differentiated ITSCs after bag-culture. ALP, alkaline phosphatase; ITSCs, inferior turbinate stem cells.

Investigating potential changes in the stemness characteristics of ITSCs after bag cultivation, we applied a directed osteogenic differentiation assay by exposing the cells to dexamethasone, β-glycerophosphate and L-ascorbic acid-2-phosphate (see Figure 3A for schematic overview). We assessed the potential activity of ALP, a hallmark of early osteogenic differentiation, after nine days of differentiation. Bag-cultured ITSCs exhibited ALP activity (Figure 3B, left panel) comparable to three-dimensionally pre-cultivated ITSCs (data not shown), while control cells exposed to FCS only remained ALP negative (data not shown). ITSCs pre-cultivated in bags revealed calcium deposition after 23 days of directed differentiation, as shown by Alizarin Red S staining (Figure 3B, middle panel), indicating late osteogenic differentiation. Von Kossa staining further substantiated mineralization of three-dimensional pre-cultivated (data not shown) and bag-cultured ITSCs (Figure 3B, right panel). On the contrary, control cells cultivated in FCS-containing medium without additional factors for 23 days displayed no signs of mineralization (data not shown).

Analyzing osteogenic differentiation of bag-cultured ITSCs in more detail, we assessed potential expression of respective osteogenic markers via reverse transcription PCR. Differentiated ITSCs, which had been three-dimensional cultured before differentiation, showed the message of characteristic osteogenic markers osteopontin and osteocalcin, while no expression was detectable in undifferentiated ITSCs cultivated as neurospheres. Notably, ITSCs previously cultivated in culture bags also expressed osteocalcin and osteopontin after differentiation, further suggesting the presence of osteogenic cell types (Figure 3C).

### ITSCs give rise to adipogenic cell types after bag-culture

In addition to osteogenic differentiation, we determined the capability of bag-cultured ITSCs to give rise to adipogenic cell types in a directed differentiation assay. After 30 days of differentiation, formation of Oil Red O-positive lipid droplets indicated successful adipogenic differentiation of bag-cultured ITSCs (Figure 3D). We observed no significant differences between the adipogenic differentiation potential of three-dimensional- and bag-cultured ITSCs (data not shown).

### Bag-cultured ITSCs maintain their ability to differentiate into neuronal cells

**Figure 4:**
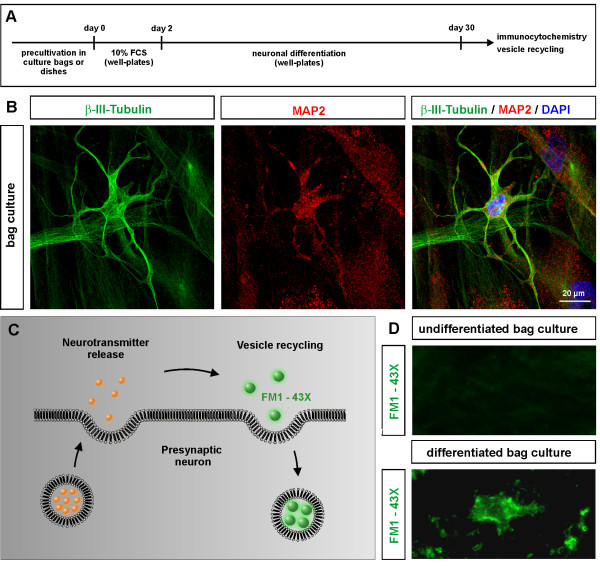
**ITSCs maintain neuronal differentiation capacity after bag culture. (A)** Schematic overview of directed neuronal differentiation of ITSCs. **(B)** Representative immunocytochemical analyses (biological triplicate) showed coexpression of neuronal markers β-III-tubulin and MAP2 in bag-cultured ITSCs after four weeks of directed neuronal differentiation. **(C)** Schematic view on synaptic vesicle recycling assay. **(D)** Synaptic vesicle recycling assay revealed no FM1-43FX signal in undifferentiated ITSCs (upper panel), while bag-cultured ITSCs differentiated for 30 days were able to recycle synaptic vesicles, as visualized by FM1-43FX-labeling (lower panel). Representative images of biological triplicate. ITSCs, inferior turbinate stem cells; MAP2, microtubule-associated protein 2.

In order to assess their potential for neuronal differentiation, ITSCs previously cultivated in culture bags were exposed to directed neuronal differentiation conditions for four weeks (Figure 4A). Differentiated ITSCs revealed characteristic neuronal morphology accompanied by co-expression of the neuronal markers MAP2 and β-III-tubulin, as shown by immunocytochemistry (Figure 4B). The capacity of bag-cultured ITSCs to give rise to neuronal cell types was found to be comparable to three-dimensionally pre-cultivated ITSCs (data not shown).

In addition, the functionality of neuronally differentiated ITSCs was investigated via a synaptic vesicle recycling assay using the FM1-43FX dye (Figure 4C). We applied undifferentiated bag-cultured ITSCs as a negative control, which demonstrated no labeling above background level after KCl-dependent stimulation (Figure 4D, upper panel). On the contrary, ITSCs cultivated in bags followed by differentiation for four weeks demonstrated active vesicle uptake, indicating the presence of at least partly functional neurons (Figure 4D, lower panel).

## Discussion

This study describes, for the first time, the suitability of Afc-FEP cell culture bags for the cultivation of human neural crest-derived ITSCs growing within a human BP-derived fibrin matrix. We have demonstrated that ITSCs maintain their proliferative capability, vitality, expression profile, genetic stability and telomerase activity during their expansion within the bags. After bag-culture, ITSCs were able to give rise to osteogenic, adipogenic and neuronal cell types.

Residing in the adult human body, neural crest-derived stem cells (NCSCs) can be isolated from a broad range of tissues, particularly including dental pulp, periodontal ligament, palate and skin as well as oral, respiratory and olfactory mucosa [10,19-25]. NCSCs are promising candidates for regenerative medicine [25] as they are easily accessible and expandable *in vitro*, and maintain robust differentiation capabilities [20,26-29]. Application of NCSCs in therapeutic approaches further offers the possibility to transplant them autologously into their donor, while additionally avoiding ethical issues. As the low amount of NCSCs within their respective niches of the adult human body limits their direct use for transplantations, *in vitro* expansion remains as a vital alternative. Such necessary cultivation procedures greatly increase the risk of transmitting infectious agents [2], such as viruses [30,31] or bacteria [3] together with the transplant. In this regard, the FDA, as well as respective bodies of the European Union (Euro GMP guidelines), prescribe good tissue handling practices in a sterile environment with minimal contamination risk as well as recommend against the use of animal-derived sera [5,12,32].

In terms of avoiding the application of animal sera, we recently reported the suitability of a human BP-derived fibrin matrix for cultivation of ITSCs. While a fibrinous nanofiber network allowed their three-dimensional growth and fast proliferation, ITSCs were shown to maintain inherent stemness characteristics, including the ability to give rise to mesodermal and ectodermal cell types [11]. In addition, application of human BP presents the possibility of autologous and personalizable ITSC expansion, as ITSCs and BP can be derived from the same patient.

Next to avoidance of animal-derived sera for stem cell cultivation, clinical-grade sterile culture conditions must be assured. These conditions are practically limited to cost intensive cleanrooms with airlocks which require highly trained personnel. In contrast to cleanrooms, application of the Afc-FEP cell culture bags used here is cost-reducing and allows easy handling, while luer ports allow sterile docking to a completely closed culture system. Expansion of cells using Afc-FEP culture bags minimizes the risk of virus infections or bacterial contamination; this was demonstrated by Luhm *et al*. for large-scale generation of natural killer lymphocytes [33]. In addition, Krause and coworkers used 250 ml cell culture bags to cultivate peripheral blood lymphocytes for treatment of colon and lung cancer patients in a clinical phase I trial [7]. Further demonstrating the suitability of Afc-FEP bags for clinical grade cell cultivation, Lima *et al*. showed successful bag culture of cord blood cells followed by their transplantation into ten patients with advanced hematological malignancies [6]. Notably, expansion within culture bags was also reported to be feasable for bone marrow cells for three weeks before intramyocardial transplantation. Retransplanted cells caused no infusion-related toxicities, such as allergic reactions, respiratory distress or hypertensive responses [8], suggesting the suitability of the future application of Afc-FEP bags, which were used for ITSCs cultivation in the present study, for cell expansion prior to transplantation.

## Conclusions

In summary, we demonstrate here, for the first time, the successful cultivation of neural crest-derived human stem cells within clinical GMP-graded Afc-FEP bags using a human BP-supplemented medium. Apart from assuring their vitality and genetic stability, our findings emphasize the unchanged differentiation capability of the cultivated NCSCs, particularly including the ability to give rise to osteogenic and adipogenic, as well as neuronal cell types.

**Figure 5:**
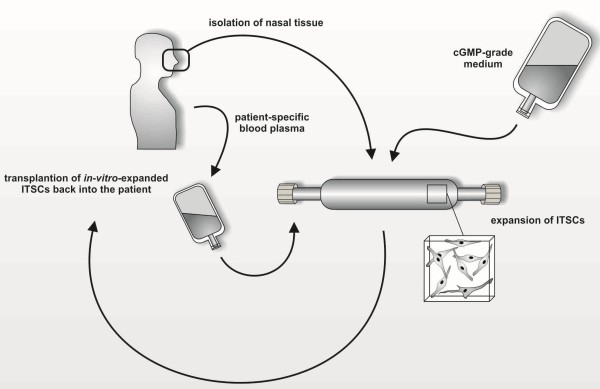
**Schematic view of potential clinical application of ITSC bag culture.** Isolation of ITSCs and patient-specific blood plasma may be followed by expansion of ITSCs within Afc-FEP cell culture bags containing cGMP-grade medium supplemented with 10% autologous blood plasma. Cultivation within cell culture bags may be directly followed by transplantion of ITSCs back into the patient. ITSCs, inferior turbinate stem cells.

Given these promising findings, we suggest that there is great potential of the presented bag system for cultivation of ITSCs for future clinical applications. As schematically depicted in Figure 5, ITSCs may be isolated via minimally invasive surgery, and BP can be derived from the same patient. Subsequent expansion of ITSCs may be performed within Afc-FEP cell culture bags using FDA-approved cGMP-grade medium supplemented with autologous blood plasma. Expanded ITSCs may be directly transplanted back into their donor, representing a potentially valuable clinical procedure for treating severe craniofacial injuries or damaged *nervus facialis*.

## Abbreviations

(D)MEM: (Dulbecco’s) modified Eagl’s medium; AB: antibody; Afc: American Fluoroseal Corporation; ALP: alkaline phosphatase; BP: blood plasma; cGMP: Clinical Good Manufacturing Practice; FCS: fetal calf serum; FDA: Food and Drug Administration; FEP: fluorinated ethylene propylene; GFP: green fluorescent protein; HBV: hepatitis B virus; HCV: hepatitis C virus; ITSCs: inferior turbinate stem cells; LSM: laser scanning microscopy; MAP2: microtubule-associated protein 2; NCSCs: neural crest-derived stem cells; PBS: phosphate-buffered saline; PFA: Paraformaldehyde; PI: propidium iodide

## Competing interests

The authors declare that they have no competing interests.

## Authors’ contributions

JFWG: Substantial contributions to conception and design of the study, data acquisition and analysis, including cell culture, phase contrast and confocal microscopy, transfection of ITSCs (Figure 1), RT-PCR analysis (Figure 2), telomerase activity measurement (Figure 2), graphical abstract (Figure 5), figure layout, manuscript drafting and writing. LMG: Data acquisition and analysis, including cell culture (proliferation, vitality, DNA-content analysis, Figure 2), mesodermal and ectodermal differentiation assays (Figures 3 and 4), manuscript drafting and writing. JM: Data acquisition and analysis, including ectodermal differentiation assay (Figure 4), critically revising manuscript for important intellectual content. HS: Interpretation of the data, figure layout, critically revising manuscript for important intellectual content. DW: Substantial contributions to conception and design of the study, interpretation of the data, figure layout, critically revising manuscript for important intellectual content. CK: Substantial contributions to conception and design of the study, interpretation of the data, Figure layout, critically revising manuscript for important intellectual content. BK: Substantial contributions to conception and design of the study, interpretation of the data, figure layout, critically revising manuscript for important intellectual content. All authors agree to be accountable for all aspects of the work in ensuring that questions related to the accuracy or integrity of any part of the work are appropriately investigated and resolved. All authors read and approved the final manuscript.
